# Neuroprotective and anti-neuroinflammatory activity of frankincense in bile duct ligaion-induced hepatic encephalopathy 

**DOI:** 10.22038/IJBMS.2023.68775.14991

**Published:** 2023

**Authors:** Marziehsadat Mirshafiei, Azadeh Yazdi, Siamak Beheshti

**Affiliations:** 1 Department of Plant and Animal Biology, Faculty of Biological Science and Technology, University of Isfahan, Isfahan, Iran; 2 Department of Medicine, Najafabad Branch, Islamic Azad University, Najafabad, Iran

**Keywords:** Frankincense, Hepatic encephalopathy, Hippocampus, Neuroprotection, Tumor necrosis factor alpha

## Abstract

**Objective(s):**

Hepatic encephalopathy induces cognitive disturbances. Patients show neuroinflammation due to accumulation of toxic substances. Frankincense has neuroprotective and anti-inflammatory properties. Accordingly, we intended to evaluate the impact of frankincense on memory performance, inflammation, and the amount of hippocampal neurons in bile duct-ligated rats.

**Materials and Methods:**

The bile duct was ligated in three groups of adult male Wistar rats (BDL groups). In two of these groups, frankincense was administered (100 or 200 mg/kg; by gavage) starting from one week before surgery to 28 days after surgery. The third BDL group received saline. In the sham group, the bile duct was not ligated and the animals received saline. Twenty-eight days after surgery, spatial memory was evaluated by the Morris water maze test. Five rats from each group were sacrificed to measure the expression of the hippocampal tumor necrosis factor-alpha (TNF-α). Three rats from each group were perfused to determine the amount of hippocampal neurons.

**Results:**

Bile duct ligation impaired memory acquisition, while frankincense amended it. Bile duct ligation significantly increased the expression of TNF-α. Frankincense reduced TNF-α in BDL rats, significantly. The number of neurons in the hippocampal CA_1_ and CA_3_ areas was significantly lower in the BDL group and in the group that received frankincense (100 mg/kg) equated to the sham group. Frankincense (200 mg/kg) augmented the amount of neurons in the CA_1_ area, slightly and in the CA_3_ area, significantly.

**Conclusion:**

The results indicate the anti-inflammatory and neuroprotective effects of frankincense in bile duct ligation-induced hepatic encephalopathy.

## Introduction

The liver is a crucial structure that clears the majority of endogenous and exogenous toxins (1). Liver failure leads to accumulation of these toxic compounds, which may spread to the brain and disturb its performance (2). Ammonia is a central contributor to these cerebral alterations (3). Hepatic encephalopathy (HE) is defined as the modifications in brain function that result from liver failure (4). For studying HE in prolonged liver disease, bile duct ligation (BDL) is utilized (5). BDL in rodents leads to fibrosis, cholestasis, and portal inflammation. As rats do not have a gall bladder, they are mainly adapted to the BDL model (6). In order to produce the BDL model, the common bile duct is ligated binary with or without transection at the center between the two ligatures (7, 8). BDL causes neuroinflammation that leads to memory deficiency in rats with hepatic encephalopathy (9).

The Boswellia species (Burseraceae) produce a gum resin that is recognized as frankincense. It has potential anti-inflammatory properties (10, 11). For example, it showed an anti-neuroinflammatory activity by decreasing the hippocampal TNF-α level in the LPS-treated rats (12). In addition, it did not show any significant side effects or toxicity (13). 

Studies suggest that bile-duct ligation affects the hippocampus in rats (14). It is well-known that the hippocampus has an essential role in memory formation (15). Due to the detrimental effects of bile duct ligation on memory formation and because of the potential anti-inflammatory and neuroprotective activity of frankincense, we aimed to appraise the effect of chronic administration of frankincense on spatial memory formation, levels of the inflammatory cytokine TNF-α, and the amount of hippocampal neurons in bile duct-ligated rats.

## Materials and Methods

***Animals***

The study was performed on laboratory animals. Thirty-two adult male Wistar rats (300 ± 20 g), reproduced in the animal facility of the Faculty of Biological Science and Technology, University of Isfahan were used. The standard protocol of animal keeping conditions was consistent with the guidelines of Iran National Committee for Ethics in Biomedical Research. Rats were held in standard polycarbonate cages (42 cm × 26.5 cm × 15 cm). The animal room conditions were controlled with a 12/12 hr light/dark period (lights on at 7 a.m.) and constant temperature (20 ± 3 °C) and the animals had unrestricted admittance to food and water. The ethics committee of the University of Isfahan (Code No. IR.UI.REC.1399.078) approved the procedure.

***Experimental procedure***

**Figure 1:**
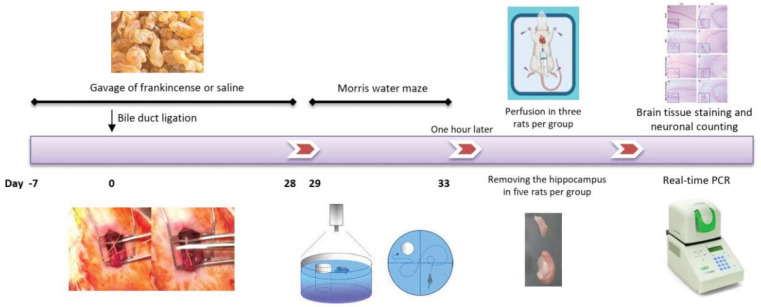
Illustration of the experimental procedure. Rats were distributed in four groups (n=8)

Animals were divided into four groups (n=8). Group 1 underwent the operation surgery without bile duct ligation. Groups 2, 3, and 4 were bile duct ligated. Groups 1 and 2 received saline and groups 3 and 4 received frankincense (100 or 200 mg/kg, by gavage) from one week before surgery to 28 days after surgery (35 days). On days 29–33 after surgery, the Morris water maze (MWM) test was performed to evaluate spatial memory formation. One hour after completion of MWM, three rats from each group were perfused and their brains were detached and stored in paraformaldehyde 4%. These brains were used for quantifying the neuron numbers. Meanwhile, the hippocampi of five rats in each group were removed and frozen immediately in liquid nitrogen. These were used for quantification of the hippocampal TNF-α (Figure 1).

***Bile duct ligation surgery***

Bile duct ligation surgery was done as previously described with some modifications (16). Twelve hours before the surgery, the animals were food restricted. Ketamine (100 mg/kg; IP) was used for anesthesia and xylazine (10 mg/kg; IP) as a sedative. In the sham group, the bile duct was manipulated, but not ligated. The abdominal fur of the rats was shaved, and the skin was sterilized with betadine. The abdomen was opened by a 2 cm long incision. Then, the peritoneal cavity was opened. The liver was raised with soaked (saline solution) cotton to visualize the hilum. The bile duct was exposed by moving the gut caudally, and its round was sutured with a 5-0 silk thread. A second ligation was added with a one-centimeter distance. Then the bile duct in between was dissected. In order to replace the abdominal organs in their positions, 0.5 ml saline was applied to the peritoneal cavity. The peritoneum and skin were stitched with 5–0 silk and disinfected with betadine. Finally, each rat received 1 ml saline (IP), and let recover.

***Preparation of the aqueous extract of frankincense***

Preparation of the aqueous extract of frankincense was performed as reported earlier (17). In brief, frankincense was crushed and waterlogged in distilled water. After 24 hr, it was warmed in a 50 °C water bath for 60 min and sieved before injection. The doses of frankincense (100 and 200 mg/kg) were designated consistent with studies indicating its usefulness in cognitive functions (18).

***Quantification of the hippocampal TNF-α***

The mRNA levels of the hippocampal TNF-α were measured by the real-time PCR, as previously defined (19). In brief, the right hippocampi of five rats per group were pounded completely. It was then mixed with 250 μl cold phosphate-buffered saline. Extraction of the total RNA was done by the RNX-PLUS solution (SinaClon, Iran). For lysing the probable genomic DNA, 1 U RNase-free DNase I (Thermo Fisher Scientific Inc., United States) was exploited. The extracted RNAs were quantified and qualified using a NanoDrop spectrophotometer (Thermo Scientific, USA). One microgram of the total RNA was used to make complementary DNA (cDNA). The cDNA synthesis was done, according to the manufacturer′s guidelines (Addbio, South Korea). The housekeeping gene was β-actin. Primers were designed by means of the NCBI primer design tool (Table 1). The Real-time quantification was performed in a reaction volume of 11 μl with 5 μl SYBR Green I Master mix (AMPLIQON, Denmark), forward and reverse primers, and 10 ng cDNA. The Bio-Rad (Bio-Rad, USA) thermal cycler was used for PCR. The cycling conditions were as follows: Initial denaturation: 30 sec at 95 °C, Denaturation and annealing: 40 cycles at 95 °C for 30 sec and 55 °C for 30 sec. 

The mRNA levels were calculated using the Livak formula (20). 

***Tissue sampling and Nissl staining***

Tissue sampling and staining were accomplished as formerly described (21). In brief, three rats from each group were perfused with phosphate-buffered saline (PBS) followed by 4% paraformaldehyde. Then, the brains were detached and fixed in 4% paraformaldehyde. Brains were then embedded in paraffin and appropriate 7 μm thick coronal sections were provided from the hippocampus (the part between Bregma -2.30 to Bregma -2.54). Sections were stained using Nissl staining to measure the number of neurons in the CA_1_ and CA_3_ areas of the hippocampus. Nuclei were stained with 0.1% Cresyl Fast Violet (Merck, Germany) for 4 min. Digital images were taken from the hippocampus in three visual microscopic fields (CA_1_ and CA_3_) using 200 × magnification. All hippocampal neurons were counted in each microscopic field using the Image J software. 

***Statistical analysis***

The distribution of the data was checked by the Shapiro-Wilk test. We utilized two-way analysis of variance (ANOVA) to evaluate Morris water maze data or one-way ANOVA followed by Tukey-Kramer *post-hoc* test to evaluate TNF-α level or cell counting. Data were evaluated using the Graph Pad Prism version 9.0.1 and presented as mean ± SEM. *P*-values less than 0.05 were considered significant.

## Results

***Effect of BDL and frankincense on memory function***

**Figure 2:**
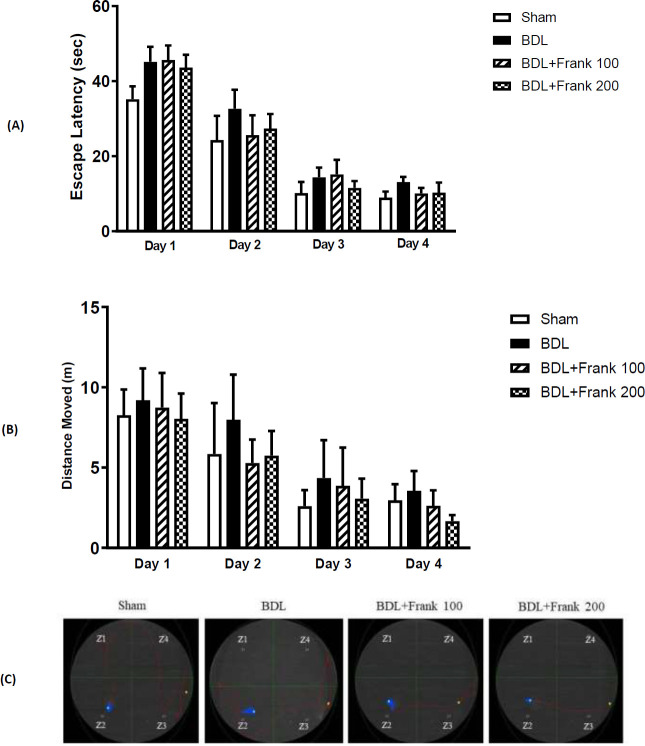
Acquisition of memory in the Morris water maze through a four-day learning procedure

Two-way ANOVA showed a significant decline in escape latencies throughout the four-day training trials in all groups, which indicates successful acquisition of memory (data not presented). Two-way ANOVA showed that the escape latencies in the final training trial did not change significantly between experimental groups (Figure 2A). Swim path traces from the final training presented in Figure 2 show that once put into the pool, the animals swam toward the platform in a short time.

**Figure 3:**
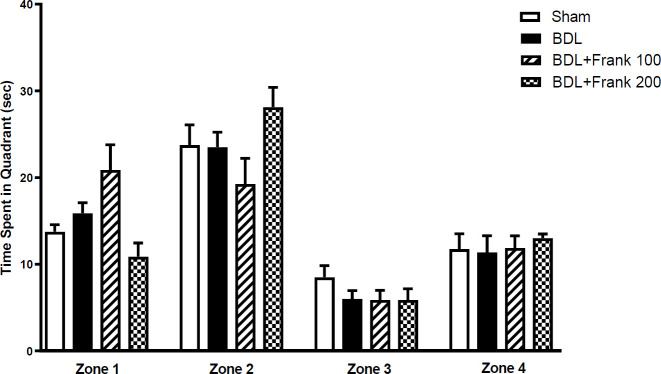
Memory retrieval in the probe trial in experimental groups

Two-way ANOVA also showed that distance movement did not change significantly in BDL rats from training trials one to four. This shows that BDL has impaired memory acquisition. However, in all the other groups there was a significant difference in distance move from training trials one to four, which shows successful memory acquisition. This shows that frankincense could improve memory acquisition in BDL rats (Figure 2B). Data analysis also showed that time spent in the target zone during the probe test did not change significantly between experimental groups. Nevertheless, there was a tendency toward increased time spent in the target zone in the BDL+Frank group compared with the BDL group (Figure 3). 

***Expression levels of the hippocampal TNF-α mRNA***

**Figure 4.**
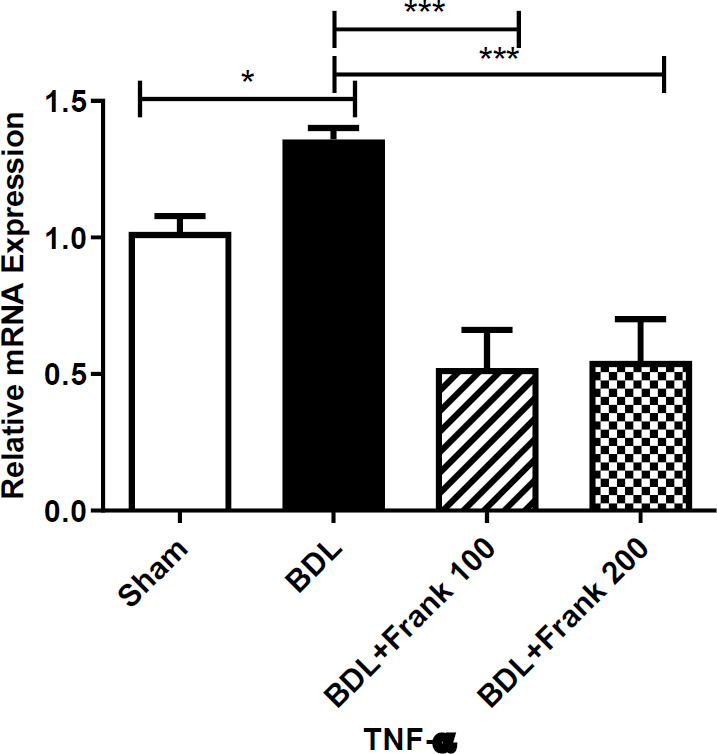
Expression levels of TNF-α mRNA in the hippocampus of rats

One-way ANOVA showed that BDL increased the levels of TNF-α mRNA in the hippocampus (*P*<0.05). Tukey-Kramer posttest showed that frankincense at both doses declined the levels of TNF-α mRNA, significantly (*P*<0.001) (Figure 4).

***Effect of frankincense on the amount of neurons in the CA***_1_ ***and CA***_3_ ***areas of the hippocampus in BDL rats***

**Figure 5:**
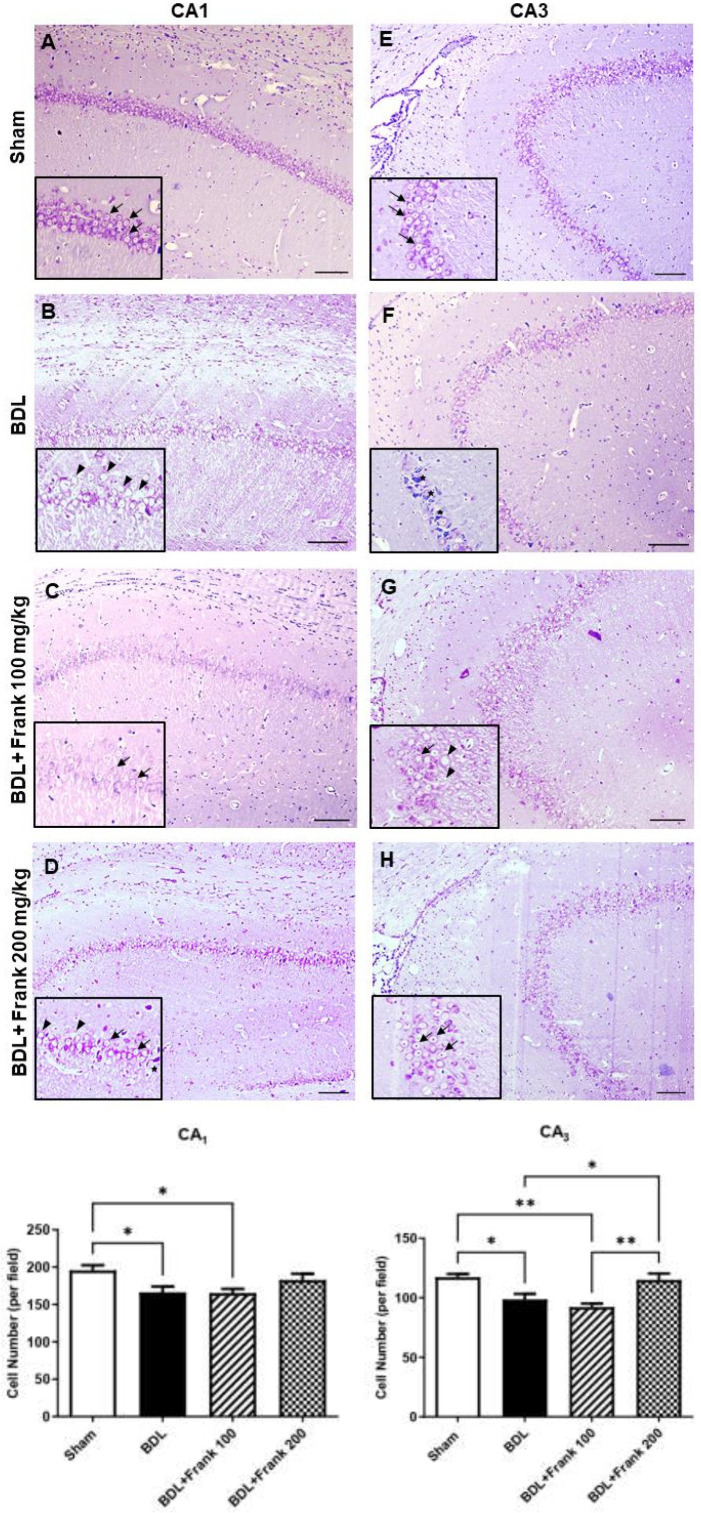
The influence of BDL on the hippocampal neuronal loss

The number of neurons in the CA_1_ and CA_3_ areas was significantly lower in the group with bile duct ligation equated to the sham group (*P*<0.05). The amount of neurons in the CA_1_ and CA_3_ areas was also significantly lower in the group that received frankincense (100 mg/kg), equated to the sham group (*P*<0.05 or *P*<0.01, respectively). Frankincense at the dose of 200 mg/kg triggered a minor increase in the amount of neurons in the CA_1_ area and significantly increased the amount of neurons in the CA_3_ area, compared with the BDL group (*P*<0.05) (Figure 5).

## Discussion

The outcomes of the current study indicated that BDL impaired memory acquisition, induced neuro-inflammation, and decreased neurons in the rat hippocampus. BDL impaired the acquisition of spatial memory but did not change spatial memory retrieval. There are controversial reports in the literature regarding the impact of BDL on memory function. It was reported that BDL impaired short-term memory, but did not change long-term memory in object recognition tests (22). On the other hand, BDL impaired memory acquisition and retrieval in the step-through passive avoidance test (23). BDL impaired spatial learning and reference memory in rats (24). It also increased oxidative stress and impaired spatial memory in developing rats (25). Minimal hepatic encephalopathy damaged learning and memory and altered structural and functional connectivity in the hippocampus (26). It is evident that BDL has the potential to affect memory function. Nevertheless, its differential impact on memory might relate to the use of different memory tasks and/or different timing regimen. 

Our results also showed that frankincense improved memory acquisition in BDL rats. Earlier studies have displayed the beneficial impact of frankincense on learning and memory, both in normal and pathological conditions (17, 18, 27-29). Administration of frankincense to pregnant rats enhanced spatial memory in the offspring (18). Frankincense facilitated the acquisition of spatial memory (30) and improved spatial memory retrieval (27). Chronic injection of frankincense amended streptozotocin-induced dementia in a time-dependent manner (17). Frankincense amended memory performance through the development of the rat brain by up-regulating calcium/calmodulin kinase II-α in the hippocampus (18). The improving impact of frankincense on memory acquisition in BDL rats might be related to all of its potential properties for enhancement of memory function. 

Our results indicated that BDL up-regulated the mRNA levels of the hippocampal TNF-α, which indicates a neuroinflammatory condition. In this regard, earlier studies have indicated that BDL augmented the levels of TNF-α in plasma, and the mRNA and protein levels of TNF-α in the hippocampus and cortex (31). Meanwhile, frankincense declined the levels of hippocampal TNF-α. This can be explained based on the anti-inflammatory activity of frankincense. Extended studies have proved the anti-inflammatory activity of frankincense or its ingredients (12, 32-36). For example, frankincense reduced the hippocampal TNF-α levels in the LPS-treated rats (12). Pre-treatment with incensole acetate, a crucial ingredient of frankincense reduced TNF-α and amended LPS-induced learning and memory impairments in rats (33). Also, incensole acetate decreased glial activation and repressed the expression of TNF-α mRNA in the mice brain traumatic area exposed to closed head injury (37). Therefore, it can be presumed that frankincense has declined TNF-α levels in the hippocampus of BDL rats and thereby reduced neuro-inflammation and improved memory acquisition. 

In the current study, we also showed the neuroprotective role of frankincense in the hippocampus of BDL rats. The number of neurons in the CA_1_ and CA_3_ areas of the hippocampus was lower in the group with bile duct ligation and in the group that received frankincense (100 mg/kg). Frankincense at the dose of 200 mg/kg triggered a minor rise in the amount of neurons in the CA_1_ area and significantly amplified the amount of neurons in the CA_3_ area. Various studies have confirmed the neuroprotective impact of frankincense or its major constituents. Frankincense suppressed the neuronal necrosis and behavioral alterations caused by fipronil by inhibiting the oxidative/inflammatory/apoptotic pathways (38). Frankincense and 3-acetyl-1 1-keto-β-boswellic acid (AKBA) reduced neuronal cell death induced by oxygen, glucose, and serum deprivation (OGSD) by reducing oxidative stress. Frankincense also protected against glutamate-induced oxidative stress and apoptosis in PC12 and N2a cells (39). Incensole acetate suppressed neurodegeneration of the hippocampus and reduced neurological severity scores and amended cognitive ability in object recognition tests after closed head injury (37). Boswellic acids taken by rats with rotenone-induced parkinsonism exhibited improved general motor performance, reduced inflammatory markers, and augmented levels of dopamine in the striatum (40). Taken together, it seems that frankincense shows neuroprotective activity in the hippocampus of the BDL rats by reducing neuroinflammation.

Group 1 underwent the surgery operation without bile duct ligation. Groups 2, 3, and 4 were bile duct ligated

Groups 1 and 2 received saline and groups 3 and 4 received frankincense (100 or 200 mg/kg, by gavage) from one week before surgery to 28 days after surgery (35 days). On days 29–33 after surgery, the Morris water maze (MWM) test was accomplished to evaluate spatial memory formation. One hour after MWM, three rats from each group were perfused. Their brain tissues were used for quantifying the neuron numbers. The hippocampi of five rats in each group were removed and frozen immediately in liquid nitrogen. These were used for quantification of the hippocampal TNF-α

**Table 1:** Primers used for amplification of the housekeeping and the target genes, and the related amplicon sizes

**Target gene**	**Sequences ** **(5'→3')**	**Amplicon size **
**β-actin**	Forward: CTGTGTGGATTGGTGGCTCT Reverse: CAGCTCAGTAACAGTCCGCC	135bp
**TNF-α**	Forward: AGAACTCAGCGAGGACACCA Reverse: CTGGCTGGTTGCTTGCTTTT	97bp

(A) Points designate average escape latencies or (B) distance moved by the animals. (C) Typical swim path traces from the final training. Two-way ANOVA was utilized to evaluate the data

The memory retrieval was measured by the time expended in the target quadrant. Data were signified as mean ± SEM of 8 animals per group. Two-way ANOVA was utilized to evaluate the data. No significant change occurred in the time spent in the target quadrant in experimental groups

One-way ANOVA followed by the Tukey-Kramer *post hoc* test was utilized to evaluate the data. The hippocampal TNF-α levels up-regulated in BDL rats in a significant manner. Frankincense at both doses decreased TNF-α levels, significantly. Data were shown as means ± SEM. (*P<0.05; ***P<0.001)

(A-H) Nissl staining revealed that the quantity of neurons was decreased in BDL rats compared to the sham group. (I) Quantitative examination of cell number. Frankincense reduced cell loss in the hippocampal CA3 area in a significant manner, compared to the BDL group. Data were shown as means ± S.E.M. and evaluated by one-way ANOVA followed by Tukey post-test. *P<0.05; ** *P*<0.01. : Normal cell bodies of the neurons; : tissue infiltration; *: apoptotic cells. Scale bar: 50 μm, n = 3. BDL=Bile duct ligation

## Conclusion

Our results showed that BDL impaired memory acquisition, which was associated with increased levels of TNF-α and neuronal loss in the hippocampus. In addition, frankincense showed powerful anti-neuroinflammatory and neuroprotective activity in BDL-induced hepatic encephalopathy. 

## Authors’ Contributions

S B designed the experiments; M M and A Y performed the experiments and collected data; M M, A Y, and S B discussed the results and strategy; S B and AY Supervised, directed, and managed the study; M M, AY, and S B approved the final version to be published.

## Conflicts of Interest

The authors declare no conflicts of interest.
